# Hyper-Activated Brain Resting-State Network and Mismatch Negativity Deficit in Schizophrenia With Auditory Verbal Hallucination Revealed by an Event-Related Potential Evidence

**DOI:** 10.3389/fpsyt.2020.00765

**Published:** 2020-08-03

**Authors:** Qiaoling Sun, Yehua Fang, Xuemei Peng, Yongyan Shi, Jinhong Chen, Lifeng Wang, Liwen Tan

**Affiliations:** ^1^ Department of Psychiatry, Mental Health Institute of the Second Xiangya Hospital, Central South University, Changsha, China; ^2^ Department of Clinical Psychology, Zhuzhou Central Hospital, Zhuzhou, China; ^3^ Department Psychology, Xiangtan Central Hospital, Xiangtan, China; ^4^ Department of Sleeping Disorders & Neurosis, Brain Hospital of Hunan Province, Changsha, China; ^5^ Department of Clinical Psychology, The Third Xiangya Hospital of Central South University, Changsha, China

**Keywords:** schizophrenia, auditory verbal hallucination (AVH), event-related potential, mismatch negativity (MMN), resting-state network (RSN)

## Abstract

Schizophrenia is a holergasia with unclear mechanism and high heterogeneity. Auditory verbal hallucination (AVH) study might help in understanding schizophrenia from the perspective of individual symptoms. This study aimed to investigate the activities of the resting-state networks (RSN) in the electroencephalogram (EEG) and mismatch negativity (MMN) in task-related state of schizophrenia patients with AVH. We recruited 30 schizophrenia patients without any medication for more than 4 weeks (15 AVH patients and 15 Non-AVH patients) and 15 healthy controls. We recorded the EEG data of the participants in the resting-state for 7 min and the event-related potential (ERP) data under an auditory oddball paradigm. In the resting-state EEG network, AVH patients exhibited a higher clustering coefficient than Non-AVH patients and healthy controls on delta and beta bands and a shorter characteristic path length than Non-AVH patients and healthy controls on all frequency bands. For ERP data, AVH patients showed a lower MMN amplitude than healthy controls (*p* = 0.017) and Non-AVH patients (*p* = 0.033). What’s more, MMN amplitude was positively correlated with clustering coefficient, and negatively correlated with characteristic path length on delta, theta, beta and gamma band in AVH patients. Our results indicate that AVH patients showed a hyper-activity in resting-state and may have impaired higher-order auditory expectations in the task-related state than healthy controls and Non-AVH patients. And it seems reasonable to conclude that the formation of AVH may occupy certain brain resources and compete for brain resources with external auditory stimuli.

## Introduction

Schizophrenia, which typically starts between late adolescence and early adulthood, is characterised by incompatibility among thought, behaviour, emotion and the surrounding environment (1, 2). The disease has an unclear cause, long course, tendency to relapse, and poor treatment effect, which bring a serious burden to society (3). Schizophrenia has always been regarded as a whole in research; however, this approach is inaccurate owing to the heterogeneity of the disorder (4). Schizophrenia from the perspective of individual symptoms may be easily understood (5). One of the most prevalent symptoms of schizophrenia is hallucination (6), which refers to an illusory perception (7) that occurs in the absence of external stimulus and with a clear consciousness state. Auditory verbal hallucination (AVH) involves the perception of speech in the absence of external sensory stimulation, occurs with a 50%–80% probability (8) and is the most common hallucination of schizophrenia. The study of independent AVH may be conducive to understanding schizophrenia (9).

In order to explore how AVH occurs spontaneously from the brain’s intrinsic activity. Amount works had been done by studying the brain in its so-called “resting state,” which refers to the intrinsic patterns of brain activity that are observable in the absence of an external task (10). Many early studies have found increased resting activity in the upper or middle temporal lobe in the presence of AVH (11). Dierks compared activation level of resting-state in the same schizophrenia patients group on and offset AVH and found that the activation level of auditory cortex in schizophrenia patients on AVH was increased (12). Allen et al. observed that the secondary auditory cortex was particularly active when AVH occurred (13). In a case study involving a patient with chronic AVH, elevated activity of the right medial temporal and left superior temporal gyri without external speech was observed (14). The bilateral temporal cortex is overactive when AVH occurs in schizophrenia (15).

The mismatch negativity (MMN) is an electrophysiological response that is elicited when a sequence of identical auditory stimuli is infrequently interrupted by a stimulus that deviates from the standard stimulus along one or more dimensions, which appears to represent the automatic change detection process that occurs when an acoustic event violates expectations maintained by the active auditory trace (16, 17). Some studies showed that the decreased amplitude and prolonged latency of MMN are related to the positive symptoms of schizophrenia (18), and MMN abnormality in schizophrenia patients is mainly manifested in the decrease of amplitude (19). MMN in the fronto-temporal lobe is atypical in schizophrenia patients with AVH (20).

Spontaneous potential is a kind of electroencephalogram (EEG) recorded without stimulation. Brain network analysis is a data processing method and brain network topology can be established from EEG data (21). When stimulation is introduced, the event-related potential (ERP), an EEG recorded during the advanced cognitive processing of an object (attention, memory or judgment), is obtained (22). ERP with an excellent temporal resolution is an ideal tool for studying the cognitive function of patients with psychiatric problems and has an extremely sensitive response to cognitive processing. The latency of ERP component refers to the time interval between the stimulus point and ERP component, reflecting the speed at which the brain processes stimuli, whereas the amplitude reflects the brain’s ability to respond to stimuli and resources (such as the number of neurons initiated) devoted to it.

Amount of works had been done on resting-state or task-related state activities in schizophrenia patients with AVH. Various articles from functional magnetic resonance imaging have posited a specific link between RSN [such as default mode network (23, 24), central executive network (25), auditory, and language regions (26–28)] and AH. Few EEG/magnetoencephalography studies have examined the resting state in relation to AVH (29) and found that the occurrence of AVH has been associated with increased beta oscillations in left frontoparietal regions (30) and increased gamma-theta in frontotemporal areas (31).But inadequate AVH severity and the effects of antipsychotic drugs have not been considered in these studies (32), and it is still unclear how this hyper-activated resting-state can affect the activity of the task-related state.

In this present study, we recruited drug-free schizophrenia patients with or without AVH and age-matched healthy controls. We recorded the EEG data in the resting-state and their corresponding ERP data during the auditory oddball paradigm. We aimed to examine the activities of the resting EEG brain networks and MMN in task-related state of schizophrenia patients with AVH and explore whether there were some relations between these two states.

## Materials and Methods

### Participants

Patients were recruited from the psychiatric outpatient department of the Second Xiangya Hospital of Central South University from June to December 2014. The inclusion criteria of the patients were as follows: (a) aged between 18 to 60 years, (b) met ICD-10 criteria for schizophrenia, (c) drug-free or has washed out more than 4 weeks and (d) normal hearing and right-handed. The exclusion criteria were as follows: (a) history of head injury resulting in loss of consciousness, (b) diseases of physical illness or neurological disorders, (c) comorbid with other mental disorders and with other forms of hallucinations, (d) alcohol or drug abuse history, and (e) received electroconvulsive therapy treatment in the past year. Patients meeting the diagnostic criteria for schizophrenia, according to ICD-10, were assessed by two senior clinical psychiatrists by using the Positive and Negative Symptom Scale (33).

We divided the schizophrenia patients into AVH patients and Non-AVH patients. The AVH patients consisted of 15 patients with a current history of AVH, as evidenced by a score ≥ 3 (“mild or greater hallucinatory experiences”) on the hallucination item of the PANSS positive symptom scale and the Non-AVH patients consisted of 15 patients with no lifetime history of AVH. Healthy controls (n = 15) were recruited from the local community by advertisement. The study was approved by the local ethics committee, and written informed consent was obtained from each participant.

### Auditory Oddball Task

The program was performed using the E-prime 2.0 software. Briefly, the subjects received auditory stimulus sequences consisting of 540 standard stimuli and 60 deviant stimuli delivered randomly. MMN can be elicited by auditory oddball paradigm and is the negative waveform peaking between 100 and 250 ms after stimulation without subjective effort or subjective interference (16, 17). The processing of auditory stimuli is complex, especially the encoding of duration. If AVH and shortages of auditory resources are found, the brain is most likely to make mistakes in the encoding of duration. Therefore, this study chose duration deviation to induce MMN (34). The probabilities of hearing standard and deviant stimuli were 90% and 10%, respectively. The inter-stimulus interval was 500 ms. The stimulus was delivered binaurally through Sennheiser headphones. The subjects were instructed to watch a neutral exposition to ignore the stimuli. The auditory stimulus had a pure tone and was applied in different durations (standard: 100 ms, deviant: 50 ms). The frequency of the pure-tone stimuli was 1,000 Hz, and the loudness was 70 dB (35).

### Data Recording

EEG data were recorded with an electrode cap with Ag/AgCl electrodes at 64 scalp sites according to the modified 10–20 system of electrode placement. Vertical electro-oscillogram was recorded from one electrode fixed below the right eye. The reference electrode was at FCz. All the electrode impedances were maintained below 5 kΩ. Electrical activity was recorded by using the Brain Vision Recorder software (Germany, Brain Products) with an amplifier band pass of 0.1 and 1,000 Hz and digitised at 500 Hz. The first block of the experiment was to record the resting-state EEG data for 7 min when the participants were asked to sit quietly with their eyes closed. After the first block, the participants had a break for 1–2 min. In the second block, the ERP data were recorded when the subjects received the auditory oddball task.

### Analyses of the Resting-State EEG Data and ERP Data

We estimated Coherence (*Coh*), which is the linear relationship at a specific frequency between the two signals *x(t)* and *y(t)* on the basis of their cross-spectrum (36). In brief, *Coh* is expressed as follows,

$$Coh_{xy}(f)=\frac{|S_{xy}(f){|}^{2}}{S_{xx}(f)S_{yy}(f)}$$

*S_xy_(f)* indicates the cross-spectrum of *x(t)* and *y(t)* at the frequency *f*; *S_xx_(f)* and *S_yy_(f)* denote the autospectrum from the fast Fourier transformation on *x(t)* and *y(t)*, respectively.

Network analysis was performed using electrodes as the nodes. The coherence between electrode pairs was used to measure the interactions between two regions. After the weighted network was calculated, a threshold (0.200) that can guarantee the connection of network was used to binarize the network. Based on the binarised network, the characteristics of the network can be quantitatively denoted by the network measurements, including clustering coefficient (C) and characteristic path length (L) (21). The weighted matrix obtained from *Coh* is referred to as *w*, and *w_ij_* represents the connectivity between nodes *i* and *j*. Node number is denoted by *N,* and the set of all nodes in the network is denoted by *Ω*. For a given threshold *T*, the adjacent matrix *w* is binarized as,

$$w_{ij}=\{\begin{array}{c} 1,w_{ij}\geq T\\ 0,w_{ij}<T \end{array}$$

The threshold is 0.200. *k_i_* is the degree of the node *i* that was defined as,

$$k_{i}=\sum_{j\in \Omega }w_{ij}$$

*t_i_* is the number of triangles that can be formed between node *i* and its neighboring nodes, and was defined as,

$$t_{i}\frac{1}{2}\sum_{j,h\in \Omega }w_{ij}w_{ih}w_{jh}$$

*d_ij_* is the shortest path between node *i* and node *j*. The characteristic path length *L* for a graph can then be defined as,

$$L=\frac{1}{N}\sum_{i\in \Omega }L_{i}=\frac{1}{N}\sum_{i\in \Omega }\frac{\Sigma_{j\in \Omega ,j\neq i}d_{ij}}{N-1}$$

The clustering coefficient can be calculated as,

$$C=\frac{1}{N}\sum_{i\in \Omega }\frac{2t_{i}}{k_{i}(k_{i}-1)}$$

Five frequency bands including delta (1–4 Hz), theta (4–8 Hz), alpha (8–13 Hz), beta (13–30 Hz) and gamma (30–60 Hz), were selected for analysis.

MMN was acquired at Fz electrode, because MMN was predominantly distributed in the frontal–central area. The offline data of the second block was processed by using the Brain Vision Analyser 2.0 system (Brain Products GmbH, Germany). EEG data were referenced to the average of mastoids (TP9 and TP10). EEG signals were bandpass filtered using a 0.5–30 Hz (50 Hz notch). Eye movements and eye blinks were removed using an independent component analysis (ICA). Artefact rejection procedures were applied to all epochs (−200 ms pre-stimulus to 450 ms post-stimulus), with a baseline correction from −200 ms to 0 ms pre-stimulus. The latency and amplitude of MMN were measured. Latency refers to the time from the beginning of stimulation to the maximum negative peak between 100 and 250 ms, and its amplitude is the vertical distance from the baseline to the maximum peak.

## Statistical Analysis

Statistical analyses were carried out by using the Statistics Product and Service Solutions (SPSS18.0) software package. Cochran & Cox Approximate t-test was used in the analysis of PANSS-P3 score between the two patient groups. For the resting EEG data, repeated-measures ANOVA with group (AVH patients, Non-AVH patients, healthy controls) as between-subject variable, and frequency band (delta, theta, alpha, beta and gamma) as within-subject variable. For the ERP data, one-way ANOVA was performed. All post-hoc analyses used the Bonferroni adjustment. Statistical analyses were adjusted for variance nonsphericity using the Greenhouse-Geisser correction (37). Statistical significance was considered at *p* < 0.05. Spearman correlation analysis was applied to explore the relationship between MMN amplitude and properties of resting EEG data in AVH patients.

## Results

### Demographic Results

The demographic information for all participants is presented in **Table 1**. The schizophrenia patients and healthy controls were matched with respect to age, gender and education level. The two patient groups had no significant difference in PANSS positive symptom, negative symptom and general psychopathology. The difference in the PANSS-P3 was statistically significant between AVH patients and Non-AVH patients, and AVH patients had a higher PANSS-P3 score (*t’* = 15.08, *p* < 0.001).

**Table 1:** Demographic information for participant and trait questionnaires.

Item	AVH patients (*n* = 15)	Non-AVH patients (*n* = 15)	healthy controls (*n* = 15)	Statistic values	*p*
Age (year)	29.07 ± 5.67	27.64 ± 5.55	29.00 ± 5.16	*H* = 0.437	0.804
Gender (male/female)	10/5	11/4	10/5	*X ^2^* = 0.207	0.902
Education level (year)	13.47 ± 2.29	13.64 ± 3.53	15.69 ± 1.82	*H* = 0.161	0.923
Illness duration (month)	8.23 ± 7.93	11.09 ± 5.45	–	*Z = -1.345*	0.164
PANSS positive symptom	15.80 ± 2.83	13.73 ± 2.90	–	*t* = 1.824	0.081
PANSS negative symptom	14.47 ± 7.65	15.09 ± 7.49	–	*t* = -0.207	0.837
PANSS general psychopathology	32.07 ± 6.78	30.45 ± 7.16	–	*t* = 0.585	0.564
PANSS-P3	5.33 ± 1.11	1.18 ± 0.40	–	*t’* = 15.083	0.000

H, kruskal-wallis H test of multiple samples; X^2^, Pearson’s chi-squared test; t, independent t-test; t’, Cochran & Cox Approximate t test; W, Wilcoxon rank sum test; Values are presented as mean ± SE. Where the data were unavailable or no data, a hyphen “-” was used.

### Resting EEG

**Figure 1:**
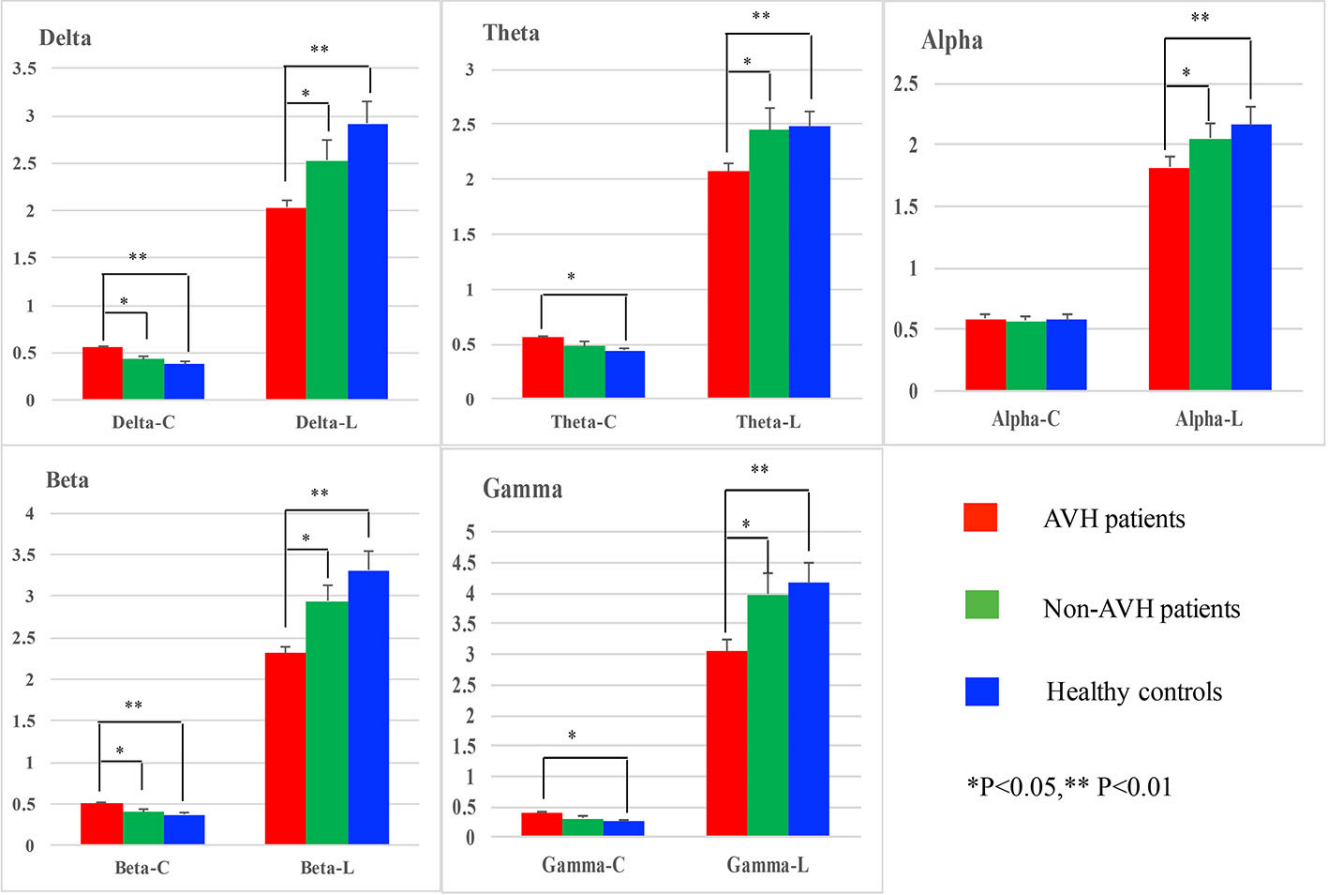
Network properties on delta, theta, alpha, beta, and gamma bands for auditory verbal hallucination (AVH) patients, non-AVH patients, and healthy controls.

For clustering coefficient, a significant interaction of group × frequency band (*F* = 2.320, *p* = 0.037) was also observed. And further simple effect analyses found that on delta and beta bands AVH patients had a higher clustering coefficient than Non-AVH patients (delta band: *p* = 0.042; beta band: *p* = 0.038) and healthy controls (delta band: *p* = 0.001; beta band: *p* = 0.002), on theta and gamma bands AVH patients showed a higher clustering coefficient than healthy controls (theta band: *p* = 0.023; gamma band: *p* = 0.019), and on alpha band, no significant difference was found among three groups. See in **Figure 1**.

For characteristic path length, main group was found (*F* = 7.754, *p* = 0.002) and post-hoc test showed that AVH patients had shorter characteristic path length than Non-AVH patients (*p* = 0.034) and healthy controls (*p* = 0.002). No interaction of group × frequency band was observed.

### MMN

**Figure 2:**
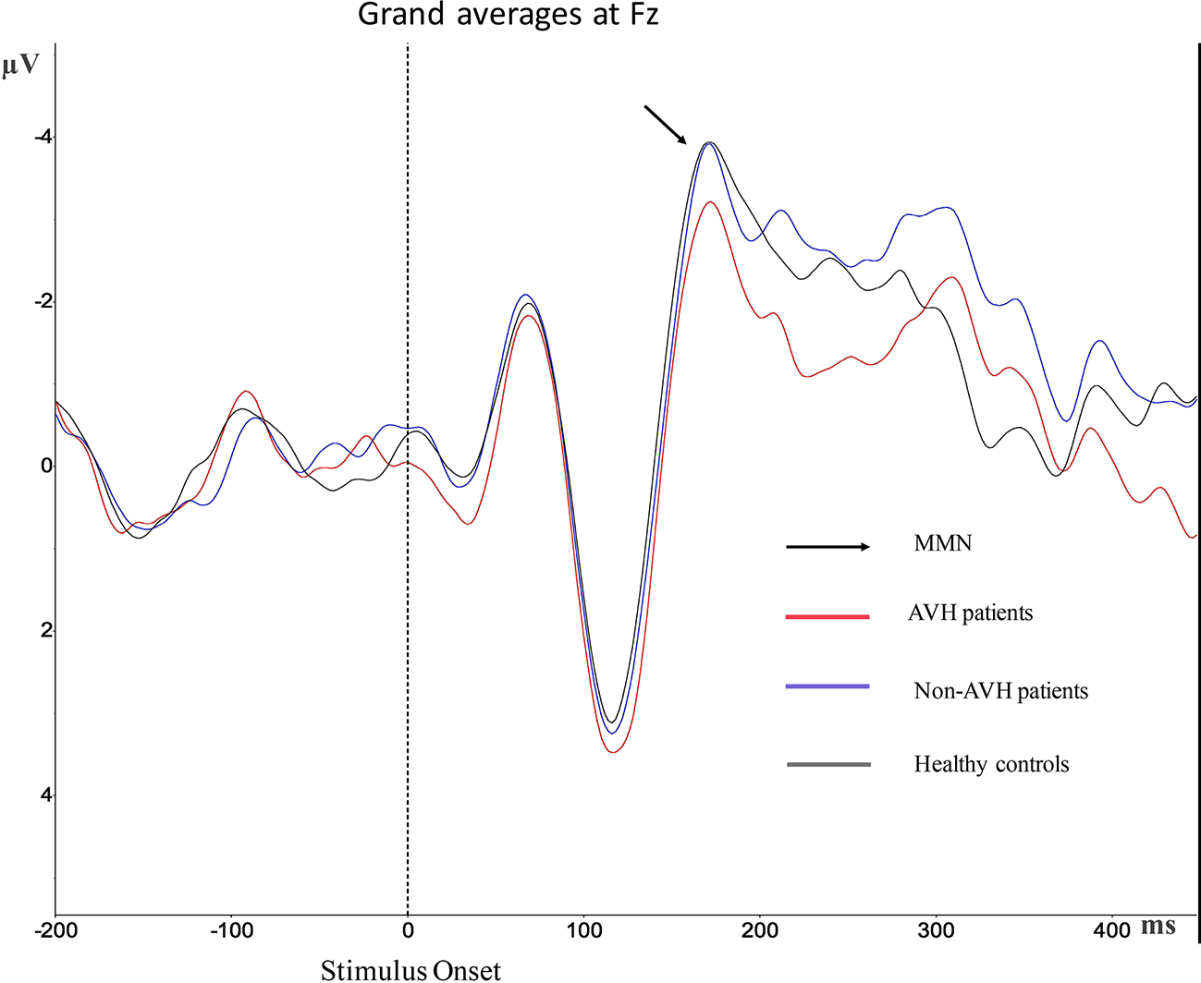
Grand average mismatch negativity (MMN) waveforms for auditory verbal hallucination (AVH) patients, non-AVH patients, and healthy controls at Fz.

Grand averages of MMN is presented in **Figure 2**. For the MMN latency, no significant differences were observed (**Table 2**). For MMN amplitude, a significant effect was found. Post hoc test revealed statistical differences between AVH patients and healthy controls (*p* = 0.017), and the MMN amplitude of AVH patients was smaller than the healthy controls. Post hoc test also revealed statistical differences between AVH patients and Non-AVH patients (*p* = 0.033), and the MMN amplitude of AVH patients was smaller than Non-AVH patients. No statistical difference was found between healthy controls and Non-AVH patients.

**Table 2:** ANOVA results for the mismatch negativity (MMN) latency and amplitude.

	AVH patients	Non-AVH patients	healthy controls	F	*p*
latency	179.20 ± 12.667	177.36 ± 14.762	177.23 ± 16.361	0.063	0.939
amplitude	2.93 ± 1.684	4.48 ± 1.290	4.56 ± 1.305	5.514	0.008

**Figure 3:**
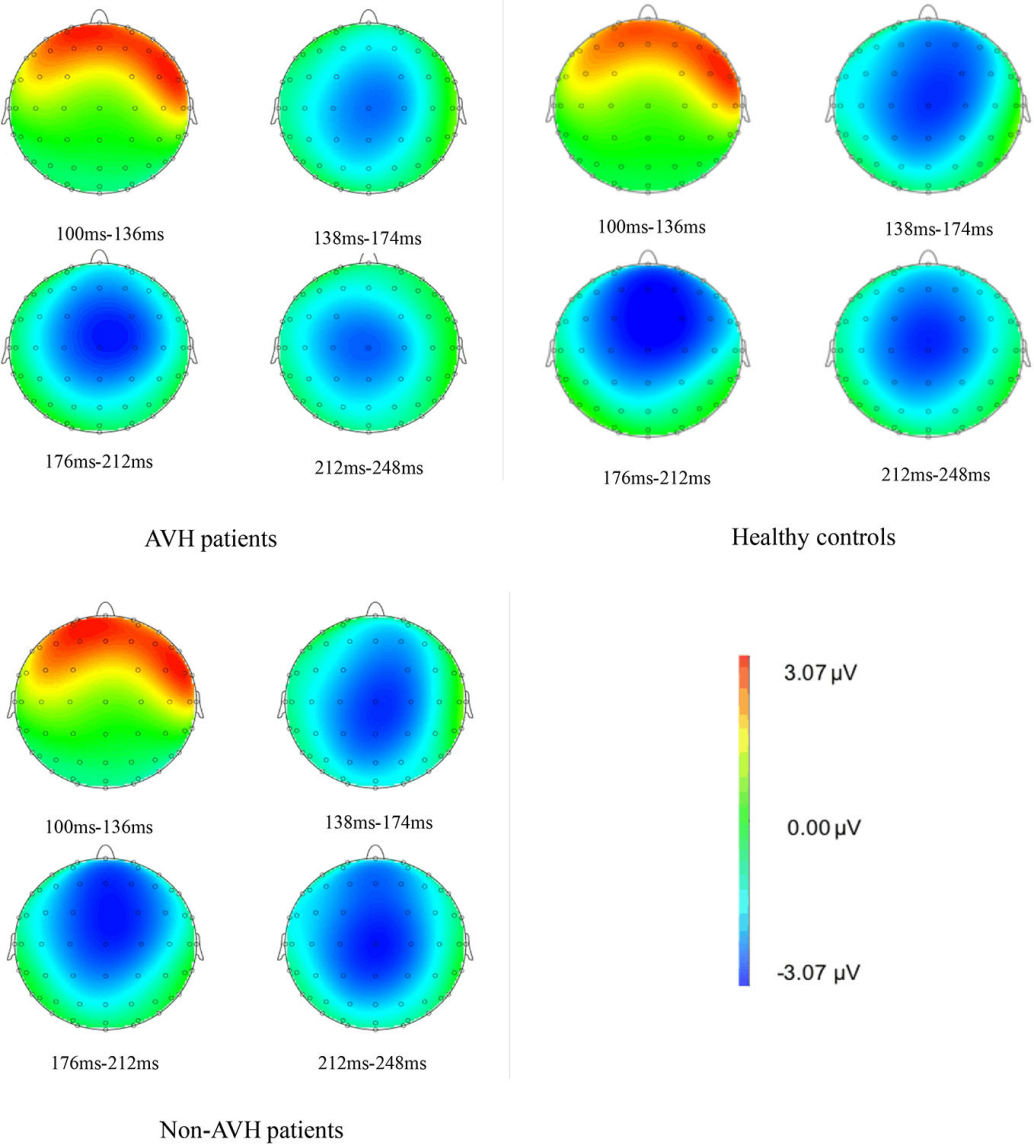
Brain topographies at Fz electrode of an interval 100–115 ms for auditory verbal hallucination (AVH) patients, non-AVH patients, and healthy controls.

The brain topographies at the Fz electrode are shown in **Figure 3**. It reflects the EEG changes from 100–248 ms after stimulation. The negative wave appeared gradually at approximately 138 ms and was increased with time. The amplitude reached the maximum during 176–212 ms and then decreased gradually. The differences between groups were obvious from 176–212 ms. The amplitude of the negative wave in AVH patients was lower than that in healthy controls and Non-AVH patients.

### Correlation Analysis in AVH Patients

Spearman correlation analysis between MMN amplitude and properties of resting EEG data in AVH patients is presented in **Table 3**. MMN amplitude was positively correlated with clustering coefficient and negatively correlated with characteristic path length in the delta, theta, beta and gamma band. Because MMN is a negative ERP component, positive loadings indicate smaller amplitudes while negative loadings indicate larger amplitude.

**Table 3:** Spearman correlation analysis between mismatch negativity (MMN) amplitude and properties of resting network in auditory verbal hallucination (AVH) patients.

	clustering coefficient					characteristic path length				
	delta	theta	alpha	beta	gamma	delta	theta	alpha	beta	gamma
MMN	.604^**^	.582^**^	.192	.571^**^	.365^*^	-.574^**^	-.570^**^	-.294	-.605^**^	-.325^*^

*Spearman correlation, p < 0.05; **Spearman correlation, p < 0.01.

## Discussion

Due to the complexity and variety of schizophrenia symptoms, as well as the interaction between symptoms, auditory-hallucination-related research is very difficult to implement. The pathogenesis of AVH in schizophrenia has not been fully understood, and no complete neurocognitive theory can explain it yet (38–40). In this study, we explored the EEG changes from the resting-state to the task-related sate in schizophrenia patients with AVH by using the ERP method. We processed the resting EEG data by brain network analysis and compared the MMN induced by auditory oddball paradigm among AVH patients, Non-AVH patients, and healthy controls. In the resting-state, we found AVH patients had a higher clustering coefficient on delta and beta band and a shorter characteristic path length on all bands than Non-AVH patients and healthy controls. In the task-related state, AVH patients showed smaller MMN amplitude than Non-AVH patients and healthy controls. What’s more, MMN amplitude was positively correlated with the clustering coefficient, and negatively correlated with characteristic path length.

The clustering coefficient evaluates the number of connections between neighbors, which provides a measure of the local structure of the network (local segregation) and an indicator of the efficiency of information transfer (41, 42). The characteristic path length evaluates the number of steps from one node to another for all possible pairs of nodes and it is usually interpreted as a metric of information integration across the overall network, which refers to the capacity of the network to become interconnected and exchange information (43, 44). The larger baseline clustering coefficient in AVH patients may reflect higher and more segregated cortical activity and the shorter characteristic path length in AVH patients may reflect more active information exchange in the resting-state brain network.

Our results are similar to previous findings. At rest, Lee et al. (30) reported greater amplitude of beta oscillations in schizophrenia patients with treatment-refractory AVH compared those without AVH, with group differences localizing to left frontoparietal regions implicated in speech and language processing. A meta-analysis demonstrated that experiencing AVH is associated with increased activity in fronto-temporal areas involved in speech generation and perception (11). In schizophrenia patients, the bilateral temporal cortex was over-activated during AVH (15). Repetitive transcranial magnetic stimulation could reduce intractable AVH by reducing the degree of activation of the left superior temporal gyrus (45). So, AVH patients may have an activated brain RSN.

In addition to schizophrenia, AVH in non-schizophrenia is also related to the activation of brain regions (46). In healthy individuals, high hallucination-prone participants reported high false alarms (i.e., reported a voice when it was not), while the temporal cortex showed high activation during these false alarms (47). To exclude the effects of delusions, negative symptoms, and antipsychotics, Remko van Lutterveld and coworkers (48) collected non-psychotic individuals with AVH to study AVHs by using resting-state fMRI. They found that in comparison with non-hallucinating controls, nonpsychotic individuals with AVH exhibit increased function in the temporal cortices (48). Therefore, AVH may be related to the activation of brain regions, and the activated brain RSN might be a neurobiological basis for auditory hallucination in schizophrenia.

In comparison with healthy controls and Non-AVH patients, AVH patients showed smaller MMN amplitude. These results are consistent with those of previous studies. AVH patients generally exhibited a lower duration MMN compared with healthy controls, particularly at F3 and Fz (49). And as measures of AVH increase, there is a corresponding decrease in MMN amplitude (50). Schizophrenia patients with clear, persistent AVH exhibited reduced MMN amplitude to duration than healthy controls and Non-AVH patients, while Non-AVH patients were not significantly different than healthy controls (4). AVH may contribute to MMN deficits in schizophrenia patients (49), and the MMN amplitude was correlated to the state and trait measures of AVH (19, 50), which further confirmed that AVH might affect the formation of MMN. MMN reflects a failure in higher-order auditory expectations (16, 17). So, AVH patients may have impaired higher-order auditory expectations than healthy controls and Non-AVH patients.

MMN was predominantly distributed in the frontal–central area, so in this study, we chose Fz electrode to extract MMN. The EEG topographies show that negative waves reached their maximum from 176–212 ms after stimulation and the largest negative wave appeared in the frontal–central area. This finding was in line with previous magnetoencephalography findings (51) and agree with the characteristic that MMN enhanced the processing in the central and frontal regions (52).

We found that AVH patients showed higher activity level in the resting-state brain network, and when external auditory stimuli occurred, AVH patients showed smaller MMN amplitude. What’s more, correlation analysis found that MMN amplitude positively correlated with clustering coefficient, and negatively correlated with characteristic path length characteristic path length in the delta, theta, beta, and gamma band. So, there may be some relation between anomalies in the two states. We speculated that the formation of AVH might occupy certain brain resources, which leads to an increased brain activation level and compete for the limited brain resources with external auditory stimuli (51, 53). Which then leads to relatively inadequate brain resources in the process of external auditory stimuli and MMN deficits. The activation of the auditory cortex in schizophrenia patients with AVH was reduced when receiving external speech (54), which might be caused by the competition between AVH and normal external speech for resources within the temporal cortex. Hubl et al. found that AVH lowered the N100 amplitude and changed the topography presumably due to a reduced left temporal responsivity (55), which indicates a competition between AVH and the normal stimuli for physiological resources in the primary auditory cortex, and that the abnormal activation of the primary auditory cortex may be a constituent of AVH (55).

This study has some limitations which need to be considered when interpreting the results. The biggest is the relatively small sample size, which limitied our statistical power to detect the smaller between-group differences and reduces the reliability of results. In this study, the values of clustering coefficient, characteristic path length and MMN amplitude in Non-AVH patients were between those in AVH patients and healthy controls. Unfortunately, no statistical difference was found between healthy controls and Non-AVH patients, which was inconsistent with previous studies (17, 56) and may be partly due to the small sample size. Increasing the sample size in future studies would provide opportunities to identify additional differences. In addition, patients collected in this study were untreated patients with serious auditory verbal hallucination. But we did not assess whether AVH actually occurred when the EEG data were recorded. This topic is the first step for us to study the specific symptoms of schizophrenia. In the future, we will further confirm our results by changing stimulus materials, enlarging the sample size, comparing the situation before and after medication, and collecting refractory AVH patients for a better understanding of the mechanism of AVH and schizophrenia.

## Conclusion

In summary, our results indicate that AVH patients showed a hyper-activity in resting-state and may have an impaired higher-order auditory expectations in the task-related state than healthy controls and Non-AVH patients. And it seems reasonable to conclude that the formation of AVH may occupy certain brain resources and compete for brain resources with external auditory stimuli.

## Data Availability Statement

All datasets presented in this study are included in the article/supplementary material.

## Ethics Statement

The studies involving human participants were reviewed and approved by Biomedical Ethics Board of the Second Xiangya Hospital. The patients/participants provided their written informed consent to participate in this study.

## Author Contributions

LT designed the study and wrote the protocol. YF managed the analyses, and QS wrote the first draft of the manuscript. XP, YS, JC, and LW contributed to conducting the study. All authors contributed to the article and approved the submitted version.

## Funding

This study was supported by the Fundamental Research Funds for the Central Universities of Central South University.

## Conflict of Interest****

The authors declare that the research was conducted in the absence of any commercial or financial relationships that could be construed as a potential conflict of interest.
